# Preparation of Conductive Cellulose Coated with Conductive Polymer and Its Application in the Detection of pH and Characteristic Substances in Sweat

**DOI:** 10.3390/ijms25126393

**Published:** 2024-06-10

**Authors:** Yujia Wu, Defa Hou, Yunwu Zheng, Xu Lin, Fulin Yang, Can Liu, Hao Sun

**Affiliations:** International Joint Research Center for Biomass Materials, Southwest Forestry University, Kunming 650224, China; wyj8099@163.com (Y.W.); houdefa001@163.com (D.H.); zyw85114@163.com (Y.Z.); linxu@swfu.edu.cn (X.L.); yangfulin0309@163.com (F.Y.); liucan@swfu.edu.cn (C.L.)

**Keywords:** electrochemical sensor, cellulose, poly (sodium 4-styrenesulfonate), conductive film, sweat detection

## Abstract

Rich biological information in sweat provides great potential for health monitoring and management. However, due to the complexity of sweat, the development of environmentally friendly green electronic products is of great significance to the construction of ecological civilization. This study utilized a simple combination of polystyrene sulfonate sodium (PSS) and filter paper (FP) to prepare cellulose materials coated with conductive polymers, developing an electrochemical sensor based on the modified materials. The mechanical and electrochemical properties of the fabricated PSS/FP membrane were optimized by adjusting the feeding dosage of PSS. The realized PSS/FP composite containing 7% PSS displayed good conductivity (9.1 × 10^−2^ S/m), reducing electric resistance by 99.2% compared with the original FP membrane (6.7 × 10^−4^ S/m). The stable current of the membrane in simulated sweat under different pH environments is highly correlated with the pH values. Additionally, when the membrane is exposed to simulated sweat with varying ion concentrations, the current signal changes in real time with the concentration variations. The response time averages around 0.3 s.

## 1. Introduction

Due to their non-invasive nature, compact size, and cost-effectiveness, flexible, wearable biosensors have garnered widespread attention in recent years. They hold tremendous potential applications in the portable and real-time detection fields [1,2,3,4,5,6,7]. Due to the rich biological information presented in sweat samples, sweat provides great potential for health monitoring and understanding the physiological and health status of human beings [8,9,10,11]. Therefore, portable biosensors that can realize sensitive, rapid, and accurate responses to various changes in sweat have become a hot research topic in the wearable sensor field.

The rapid development of wearable biosensors and bioelectronic technologies has significantly propelled the development of wearable sweat sensors [12]. Wearable sweat sensors are categorized as infrared, wired, and wireless sensors. Among them, electrochemical sensors can function as both wired and wireless sensors, holding a dominant position due to their high performance, convenience, and cost-effectiveness [13]. Flexible, wearable sensors can be applied in various scenarios. Gao et al. [14] developed a wearable, flexible integrated sensor array for electrochemical sweat sensing that is capable of simultaneously measuring the concentrations of ions and metabolites in sweat. This sensor was fabricated by attaching functional electrodes to a flexible PET and was used for the monitoring of real-time changes in analyte and electrolyte concentrations in body fluids. The sensor issued timely warnings in the case of abnormal fluid loss, showing the possibility of avoiding dehydration. However, the non-degradable nature of plastic may cause environmental pollution after the end of the usage of this PET-based sensor. Jia et al. [15] enzymatically fabricated a sweat sensor using chitosan as a substrate with flexible printing technology. The sensor was designed for non-invasive and real-time monitoring of lactate concentrations during human exercise, demonstrating high selectivity to lactate. Zhang et al. [16] designed a sensor analogous to eyeglasses, allowing for real-time extraction of sweat when it was attached to the surface of the skin, achieving non-invasive monitoring of the changes in lactate concentration in sweat. Nyein et al. [17] designed an integrated flexible, wearable device with a PET substrate, comprising integrated circuits and flexible sensing equipment, which was designed to non-invasively measure pH values and Ca^2+^ concentrations in sweat. The results showed that the prepared sensor realized high selectivity for Ca^2+^ concentration with pH decreasing from 4.4 to 6.4. Kim et al. [18] designed a flexible, wearable sensor by using screen-printing technology to assemble PB conductive carbon ink with working electrodes. This sensor collected sweat using ionophoresis technology, enabling real-time monitoring of alcohol concentration in sweat. While these sensors reported in previous literature exhibit excellent performance, they are subject to disadvantages such as complex preparation, high cost, and non-degradability.

A variety of wearable, flexible sweat sensors have been developed from degradable polymers as raw materials for the targeted detection of substances such as electrolytes [19,20,21], metabolites [22,23,24], nutrients [25,26,27], and heavy metal ions [28,29,30], and some initial applications have been achieved. To date, the most commonly used green degradable substrates for preparing sweat sensors include wrinkled paper and printing paper, but they require additional processing and/or carbonization to achieve effective sweat detection, making these protocols tedious and costly. Given this, it is still challenging to achieve high-performance and environmentally friendly sweat sensors through a low-cost and easy strategy. Current sweat sensors typically use PET substrates and incorporate internal integrated circuits to collect sweat and amplify and convert it into electrical signals. The fabrication process of these sensors is relatively complex and costly. In this study, we use low-cost, environmentally degradable cellulose substrates. By employing a simple dipping method to prepare conductive composite films, we aim to replace the complex and expensive integrated circuit processes.

In this study, commonly used filter paper (FP) was impregnated with poly (sodium 4-styrenesulfonate) (PSS) to prepare a cellulose-based conductive membrane (namely, FP/PSS). Leveraging modern analytical techniques, the effects of various feeding dosages of PSS on the mechanical properties, pH response index, and response time of the FP/PSS membrane were investigated and analyzed in detail. When 7% of the PSS was impregnated in FP, the prepared FP/PSS conductive membrane rapidly showed distinctive electrical signals corresponding to changes in pH values and ion concentrations, exhibiting high sensitivity. Therefore, benefiting from the stability and non-toxicity of the FP/PSS membrane, this study provides a novel approach for fast, sensitive, real-time, and non-destructive detection of sweat. The fabricated FP/PSS conductive membrane holds great potential for the rapid, non-destructive, and real-time online monitoring of acidity and alkalinity and shows widespread application prospects in the field of wearable electronics.

## 2. Results

### 2.1. Fabrication of Cellulose-Based Sweat Sensor

**Figure 1:**
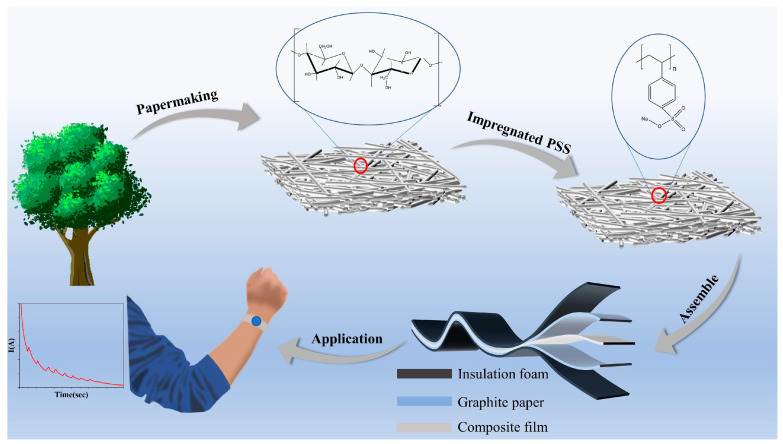
Schematic presentation of the preparation and application of PSS/FP sweat sensor.

Since it is desired to fabricate high-performance and environmentally friendly sweat sensors, this work developed a cellulose-based electrochemical sensor using a low-cost and easy impregnation strategy. As shown in Figure 1, preparing the cellulose-based electrochemical sensor involves combining FP with conductive PSS. To be specific, conductive PSS/FP membranes with different dosages of PSS were realized by immersing FP membranes into various PSS solutions. Then, a fabricated PSS/FP membrane was sandwiched between two graphite sheets to achieve the targeted sensor. The fact that the conductivity of PSS is governed by pH values and electrolyte concentration by regulating the ionization of Na^+^ suggests that pH values and electrolyte concentration also influence the electrochemical behaviors of PSS/FP membranes. In other words, our developed PSS/FP sensors exhibit possible electric responses to various pH values and electrolyte concentrations.

### 2.2. Structures and Properties of PSS/FP Membranes

**Figure 2:**
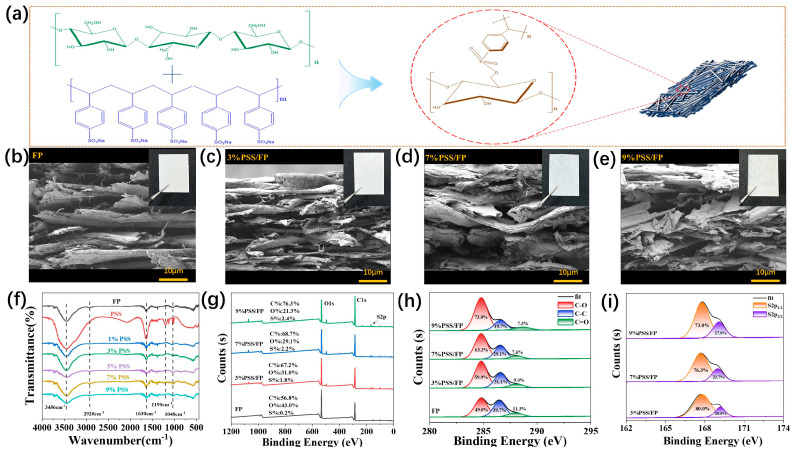
Structural characterization of PSS/FP membranes. (**a**) Schematic presentation of the interaction between PSS and FP; (**b**) SEM image of original FP (inset: digital image of FP); (**c**–**e**) SEM images of PSS/FP membranes containing different dosages of PSS (insets: digital images of corresponding PSS/FP membranes); (**f**) FT-IR spectra of FP, PSS, and PSS/FP membranes; (**g**–**i**) XPS curves of FP and PSS/FP membranes ((**g**) overall survey spectra; (**h**) high-resolution C1s spectra; (**i**) high-resolution S2p spectra).

To disclose the structures of PSS/FP membranes, the physical and chemical properties of the prepared composite films were tested. According to the chemical composition and structures of FP and PSS, there are numerous polar hydroxy (-OH) groups on the surface of the cellulose fibers of FP. Similarly, PSS contains a polar sulfonate group in each of its repeating units (Figure 2a). Therefore, it can be speculated that PSS is compatible with FP through the interaction of hydrogen bonds and sulfonate groups (Figure 2a). To confirm this speculation, the morphologies of PSS/FP membranes were characterized by SEM observation, taking the original FP as a control. A lamellar structure with overlapping voids interweaved with smooth cellulose fibers was observed in the SEM image of the original FP, and no obvious particles or coatings were found on the surface of cellulose fibers (Figure 2b). In comparison with FP, although the combination of FP and PSS did not conspicuously alter the appearance of FP according to the SEM of FP and PSS/FP membranes, muti-layered mats disappeared after loading PSS with the used impregnation method (Figure 2c–e). The addition of PSS also reduced the number of internal pores in the FP. After PSS impregnation, the degree of PSS encapsulation of cellulose fibers increased with elevated concentrations of PSS aqueous solution. Meanwhile, the internal voids in the FP were filled by PSS, and no obvious interface or peels were observed between PSS and FP. These phenomena suggest the robust interaction of PSS and FP. This uniform structure is conducive to preserving the good mechanical properties of PSS/FP membranes.

To determine the reason why PSS and FP showed excellent compatibility, the chemical interaction between PSS and FP in PSS/FP membranes was initially investigated by FT-IR measurements. As shown in Figure 2f, there are no significant differences in the basic peak shapes between FP and PSS/FP membranes. In the FT-IR spectra of all samples, the wide absorption at around 3450 cm^−1^ represents the stretching vibration of O-H bonds, mainly from the -OH groups. Interestingly, in comparison to O-H absorption (3456 cm^−1^) in the FT-IR spectrum of the original FP, the characteristic peaks of O-H bonds in the FT-IR spectra of PSS/FP membranes shifted to a low wavenumber (3442 cm^−1^), which can be ascribed to the new hydrogen bonds between FP and PSS. Moreover, the absorption near 2909 cm^−1^ corresponds to the stretching vibration of C-H bonds in PSS. Furthermore, in the FT-IR spectra of PSS, the peaks at 1175 cm^−1^ can be ascribed to the anti-symmetric vibration and symmetric vibration of S=O in sulfonic acid groups. The peak located at 1641 cm^−1^ is attributed to the frequency-doubling peak of 1,4-disubstituted benzene. The peak at 677 cm^−1^ represents the skeleton vibration of the monosubstituted benzene ring. In contrast, the new peak at 1123 cm^−1^ in the FT-IR spectra of PSS/FP membranes can be ascribed to sulfonate, suggesting a covalent interaction between PSS and FP. Therefore, the robust hydrogen bonds and sulfonate groups between PSS and FP contribute to the integral structures of PSS/FP membranes.

The chemical structures of PSS/FP membranes were also characterized by XPS measurements. In the overall XPS spectra of PSS/FP membranes (Figure 2g), the characteristic signals of C, S, and O elements were located at 284.8 eV, 169 eV, and 533 eV, respectively. The contents of S element in various membranes were 1.8% to 2.4%. Specifically, the C element was assigned to C-C, C-O, and C=O bonds according to the high-resolution C1s spectra of PSS/FP membranes (Figure 2h). In the high-resolution S2p spectra of PSS/FP membranes, two peaks at 167.8 and 168.9 eV were observed, which represent sulfone-like organic sulfurs -SO_2_Na and -SO_3_-, respectively. Thus, XPS spectra further confirmed the successful doping of PSS into the FP and the preparation of integrated PSS/FP membranes through a feasible impregnation method.

**Figure 3:**
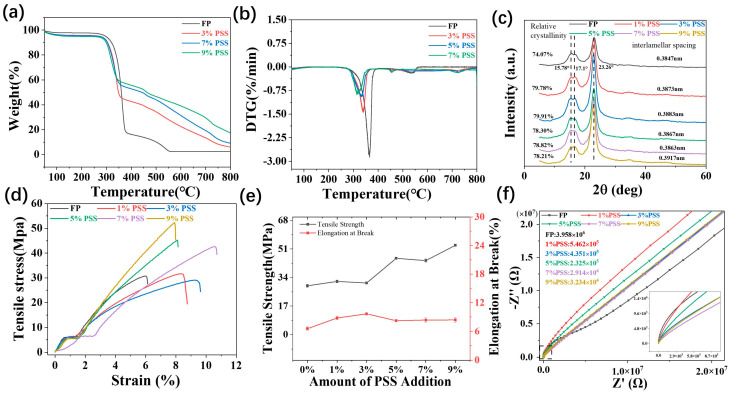
Common properties of PSS/FP membranes. (**a**) TGA curves of FP and PSS/FP membranes; (**b**) DTG curves of FP and PSS/FP membranes; (**c**) XRD patterns of FP and PSS/FP membranes; (**d**) representative stress–strain curves of FP and PSS/FP membranes; (**e**) tensile properties of FP and PSS/CP membranes; (**f**) EIS curves of FP and PSS/CP membranes.

Since thermostability is a key factor for the practical application of polymer-based materials, the thermal stability of PSS/FP membranes was evaluated through TGA (Figure 3a). PSS/FP membranes exhibited higher thermal stability than the original FP, with initial decomposition temperatures (Td) of 30~300 °C (Figure 3a). This results suggest that the addition of PSS enhanced the thermal stability of FP. The main thermal degradation of PSS/FP membranes can be separated into two stages, namely 300~360 °C and 360~800 °C. The mass loss in the temperature range of 300~360 °C was about 44.3%, which is attributed to the decomposition of the sulfonic acid group. The decomposition at 360~800 °C is ascribed to the sulfonic acid group being thermally decomposed into volatile substances. The eventual residual char ratios of PSS/FP membranes were 6.0~17.5%—higher than that of FP (2.4%). The favorable thermostability of PSS/FP membranes endows them with wide application areas. Figure 3b shows the DTG data, indicating that FP has the highest rate of mass loss, while the PSS/FP membrane exhibits a slower rate of loss.

Furthermore, crystallinity has a certain impact on the mechanical properties of cellulose-based materials. The crystalline index (CrI) of membranes was obtained through XRD. In the XRD diffraction pattern of FP (Figure 3c), the crystalline peaks at 15.78°, 17.1°, and 23.26° correspond to the (110), (110), and (200) lattice planes of cellulose I, respectively [31]. Loading PSS did not alter the crystallographic form of cellulose in FP but increased the CrI of cellulose, as verified by the ascending intensity of diffraction peaks. To be specific, the CrI values of PSS/FP membranes were 78.2~79.9%, regardless of the loading dosage of PSS, but the CrI value of FP was 74.0%. The high CrI of cellulose in PSS/FP membranes indicates the strong intermolecular interaction. This result made the PSS/FP membranes more stable at high temperatures, which is consistent with the results obtained from TGA.

The practical application of a material is closely related to its mechanical properties. To understand the mechanical performance of PSS/FP membranes, tensile tests were conducted. As shown in Figure 3d, PSS/FP membranes have a similar tensile modulus to that of the original FP. Interestingly, the addition of PSS significantly and simultaneously enhanced the tensile strength (σ) and elongation at break (ε) of PSS/FP membranes (Figure 3e). The σ and ε of the original FP were 30.7 MPa and 6.5%, respectively. After impregnating with PSS, the σ and ε of PSS/FP membranes were elevated to 53.1 MPa and 8.3%, respectively. The improvements in tensile properties were benefited by the formation of the uniform structure of PSS/FP membranes because the fiber voids in the FP were filled by PSS. The uniform structures of PSS/FP membranes can effectively disperse tensile loads, thereby improving the σ and ε of the composite membranes. However, the tensile properties of PSS/FP membranes were not found to depend on the loading dosage of PSS.

As the purpose of this work is to obtain a seat sensor, the electrochemical performance of PSS/FP membranes was measured using an electrochemical workstation (EIS). As displayed in Figure 3f, the prepared PSS/FP membranes showed similar impedance curves, consisting of a semicircle in the high-frequency region (10^3^~10^4^ Hz) and a straight line in the mid–low-frequency region (10^−3^~10^3^ Hz). The semicircle in the high-frequency region is associated with the charge transfer impedance (Rc) caused by electron gain and loss. The straight line in the mid–low-frequency region is related to solid-state diffusion processes [32]. According to well-established theory, the smaller diameter of the semicircle in the impedance curve indicates faster charge transfer rates and lower electron transfer resistance. From the fitted EIS curves (Figure 3f), it is evident that loading PSS on FP reduced the resistance and enhanced the conductivity of PSS/FP membranes. Notably, the electrochemical performance of PSS/FP membranes was determined by the concentration of PSS aqueous solutions. A PSS/FP membrane with the lowest impedance of 2.9 × 10^4^ Ω was prepared by impregnating FP with 7% PSS solution. This impedance value was reduced by 99.2% compared with original FP (3.9 × 10^6^ Ω). The impedance values of PSS/FP membranes were also decreased from 2.9 × 10^4^ Ω to 5.4 × 10^5^ Ω with an increase in the concentration of PSS aqueous solution from 1% to 7%. This phenomenon was attributed to the fact that the loading dosages of PSS in PSS/FP membranes were increased with the concentration of PSS aqueous solution (Table 1). However, using a PSS aqueous solution with a concentration of 9% resulted in a PSS/FP membrane with a resistance of 3.20 × 10^5^ Ω. This deteriorated electrochemical performance could be attributed to unfavorable impregnation caused by the high viscosity of PSS aqueous solution (Table 2). Due to the satisfactory mechanical properties and high conductivity, we believe that the PSS/FP membrane prepared with 7% PSS aqueous solution could be an ideal candidate for high-performance sweat sensors.

### 2.3. Electrochemical Performance of PSS/FP Sensor

**Figure 4:**
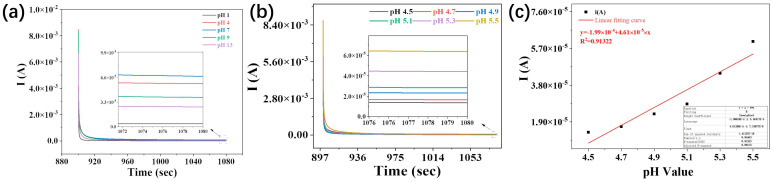
Electrochemical behaviors of PSS/FP sensor in various pH environments. (**a**) Chrono-ampere (CA) curves of PSS/FP sensor under wide ranges pH values (pH = 1~14); (**b**) CA curves of PSS/FP sensor in sweat simulant; (**c**) fitted algebraic relationship between stable current in PSS/FP sensor and pH values of sweat simulant.

After understanding its properties, the PSS/FP conductive membrane prepared with 7% PSS aqueous solution was assembled into sensors with the assistance of graphite sheets. Since the physical status of human beings is reflected in the pH values of sweat to a certain extent, the electrochemical behaviors of the assembled PSS/FP sensors with solution pH values were investigated in detail to simulate real sweat with different pH levels. To achieve this goal, different acid–base solutions with different pH values were added dropwise onto the PSS/FP sensor. As expected, in wide pH ranges, the fabricated PSS/FP sensor showed different electrochemical performance in the aqueous solutions with various pH values (Figure 4a), indicating the sensitive pH-responsive characteristics of the fabricated cellulose-based PSS/FP sensor. Under wide ranges of pH values (pH = 1~14), the conductivity of the PSS/FP sensor was improved with increasing alkalinity, and the lowest conductivity was observed after adding the solution with pH = 1 (Figure 4a). When a stable voltage of 0.01 V was loaded, the stable current values of the PSS/FP sensor were 4.1 × 10^−6^~6.7 × 10^−5^ and responded to various aqueous solutions with pH = 1~14 (Figure 4a). Although the PSS/FP sensor exhibited high sensitivity to solutions with a wide range of pH of 1~14, whether the sensor can discern real sweat remains elusive because the real pH values of human sweat are between 4.5 and 5.5. To clear this confusion, different solutions with pH values of 4.5~5.5 were used to simulate real human sweat. As shown in Figure 4b, the stable current of the PSS/FP sensor increased with increasing pH values. Taking the loading voltage of 0.01 V as a representative, the algebraic relationship between the stable current in the PSS/FP sensor and pH values was fitted, and a positive correlation between environmental pH and current values was observed (Figure 4c). The relationship between the pH values and current values for the PSS/FP sensor can be expressed by the following equation: $y=1.99\times 10^{-4}+4.61\times 10^{-5}\times x$, with a coefficient of determination (R^2^) value of 0.91322. Generally, R^2^ > 0.9 indicates a significant correlation between the independent and dependent variables. Thus, our developed PSS/FP sensor provided a unique electrochemical response to various solutions within the pH range of 4.5~5.5, making it applicable to pH monitoring of human sweat.

**Figure 5:**
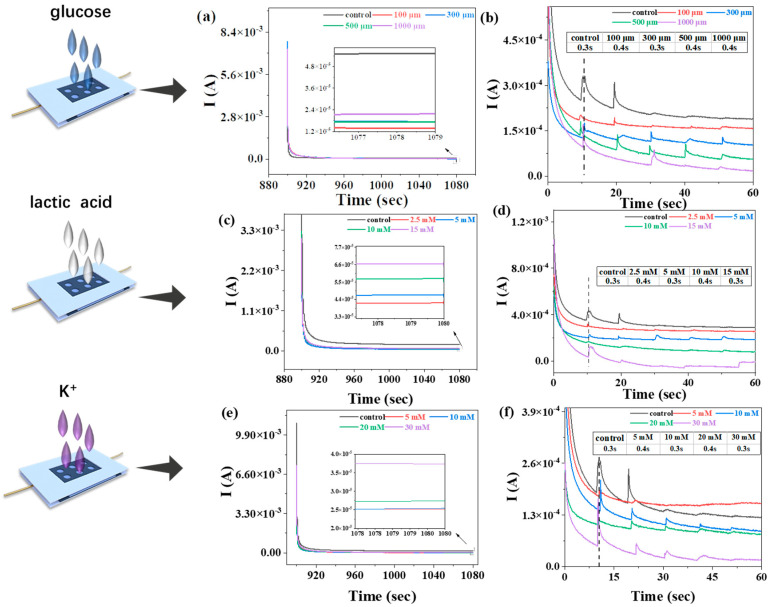
Electrochemical behaviors of PSS/FP sensor in response to concentration changes in glucose, lactic acid, and K^+^ in human sweat. (**a**) CA curves of PSS/FP sensor in response to concentration changes in glucose; (**b**) response time of PSS/FP sensor to various concentrations of glucose in artificial sweat; (**c**) CA curves of PSS/FP sensor in response to concentration changes in lactic acid; (**d**) response time of PSS/FP sensor to various concentrations of lactic acid in artificial sweat; (**e**) CA curves of PSS/FP sensor in response to concentration changes in K^+^; (**f**) response time of PSS/FP sensor to various concentrations of K^+^ in artificial sweat. Insets in (**b**,**d**,**f**) represent the detailed response time of PSS/FP sensor to concentration changes in glucose, lactic acid, and K^+^ in artificial sweat.

Glucose, lactic acid, and potassium ions (K^+^) are the major nutrients in human sweat, and their concentration changes are closely associated with various physiological and pathological states. Monitoring the variations in these nutrients can provide crucial information about body health, metabolic status, and electrolyte balance. Glucose serves as a direct indicator of body energy metabolism, lactic acid relates to exercise intensity and endurance, and changes in K^+^ concentration are closely linked to electrolyte balance and neuromuscular function. To confirm the feasibility of the PSS/FP sensor for the monitoring of nutrient concentrations in human sweat, the chrono-ampere (CA) curves of the PSS/FP sensor were obtained by the dropwise addition of artificial sweat with different concentrations of glucose, lactate, and K (Figure 5). Glucose molecules can alter the conductivity of the membrane. Higher concentrations of glucose can increase the ion migration rate in the PSS membrane, thereby enhancing the current intensity. An increase in lactate concentration leads to an increase in lactate ions and hydrogen ions in the membrane, enhancing the conductivity of the solution and increasing the current of the membrane.

According to the CA curves in Figure 5a,c,e, the fabricated PSS/FP sensor showed stable electrochemical responses to artificial sweat with different concentrations of glucose, lactic acid, and K^+^. After the dropwise addition of artificial sweat with different concentrations of glucose, the stable current values were lower than in response to the original artificial sweat. The stable current values also decreased with increasing glucose, indicating the accurate response of the PSS/FP sensor to glucose in sweat (Figure 5a). The response time of the sensor refers to the time elapsed from the addition of the test liquid to the occurrence of current fluctuations and stabilization. The response time of PSS/FP sensor to the glucose in sweat was 0.3~0.4 s (Figure 5b). Similarly, the fabricated PSS/FP sensor showed accurate responses to the concentration changes of lactic acid in sweat (Figure 5c), with a response time of 0.3~0.4 s (Figure 5d). However, after adding K^+^ dropwise, the stable current values were not all lower than for the original artificial sweat. Fortunately, the stable current values increased with increasing K^+^ concentration. Therefore, our developed PSS/FP sensor can be employed to monitor concentration changes in K^+^ in sweat (Figure 5e), with a response time of 0.3~0.4 s (Figure 5f). Due to its stable electric response and short response time, the PSS/FP sensor achieved the sensitive, rapid, and accurate detection of concentration changes in glucose, lactic acid, and K^+^ in human sweat. Based on the results and discussion presented above, we believe that our developed cellulose-based PSS/FP sensor can be used for real-time monitoring of sweat changes with high sensitivity and accuracy.

The detection limit of the membrane prepared in this experiment was 1000 μM for glucose, 50 mM for lactate, and 30 mM for K^+^ ions. Suitable concentration gradients based on the concentrations of characteristic substances in human sweat were selected for sensor testing. We used the signal-to-noise ratio method to calculate the sensor’s limit of detection (LOD). First, the stable current values of the sensor were recorded after adding blank artificial sweat and measured three times; then, the average value and standard deviation (σ_blank_) were calculated. A 3:1 ratio was chosen as the standard for the detection limit. The formula of LOD = 3σ_blank_/Slope was used to calculate the LOD. The stable current values of the three blank groups were 10.89 μA, 10.41 μA, and 10.89 μA, with a standard deviation of 0.226. The slope of the glucose calibration curve is 0.0069, and the calculated detection limit is 98.47 μM. The slope of the lactic acid calibration curve is 1.9403, and the calculated detection limit is 0.35 mM. The slope of the K^+^ calibration curve is 0.3840, and the calculated detection limit is 1.76 mM.

**Figure 6:**
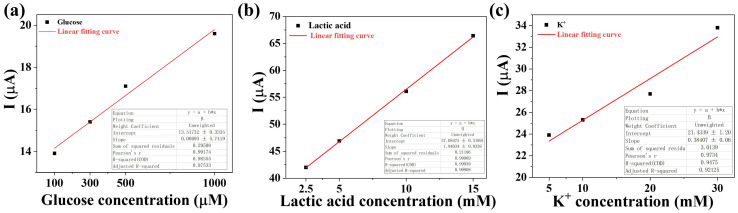
Correlation between sweat characteristic substances and stable current. (**a**) Glucose; (**b**) lactic acid; (**c**) K^+^.

The correlation coefficient (R^2^) between the stable current output of the sensor and glucose concentration after fitting was 0.88 (Figure 6a), with a lactate concentration after fitting of 0.99 (Figure 6b), and a K^+^ concentration after fitting of 0.92 (Figure 6c). After fitting, the stable current showed a high correlation with the concentration of the characteristic substances to be measured.

## 3. Discussion

This work reports a cellulose-based PSS/FP sensor for real-time sweat detection. A conductive PSS/FP membrane was prepared using a simple impregnation method, where cellulose materials were coated with PSS and sandwiched between two graphite sheets for assembly into a sensor. Due to the interaction of hydrogen bonds and sulfonate groups between FP and PSS, the achieved PSS/FP membranes showed favorable tensile properties (σ = 30.7~53.1 MPa and ε = 8.2~9.6%). In particular, the PSS/FP membrane prepared by impregnating FP with 7% PSS solution exhibited excellent conductivity, with the lowest impedance of 2.9 × 10^4^ Ω, and can be used to rapidly and sensitively monitor changes in pH values and nutrient concentrations in sweat. In summary, the developed conductive cellulose-based PSS/FP membranes hold potential for applications in the field of electrochemical sensors, in addition to providing new choices for the achievement of flexible and wearable sensors based on degradable and renewable materials.

## 4. Materials and Methods

### 4.1. Materials

Filter paper (FP) and polystyrene sodium sulfonate (PSS, 20% in water) solution were both purchased from Titan technology Inc. (Guangzhou, China) and directly used without further purification. Other chemicals, if not otherwise mentioned, were of analytical reagent grade and purchased offline.

### 4.2. Methods

A Fourier Transform Infrared (FTIR) spectrometer (iS50) was employed to investigate the chemical interactions between FP and PSS, with a resolution of 4 cm^−1^. The elemental composition of various PSS/FP membranes was measured using a Thermo Scientific K-Alpha X-ray Photoelectron Spectrometer (XPS). X-ray Diffraction (XRD) analysis was performed on a Max220 X-ray diffractometer with Cu Kα radiation. The morphologies of the prepared PSS/FP membranes were observed by a scanning electron microscope (SEM, TM3000). The tensile properties of all samples were measured with the assistance of an XLM (PC) Intelligent Electronic Tensile Testing Machine. A thermogravimetric analysis (TGA) of PSS/FP membranes was conducted on a TG 209 F3 Thermogravimetric Analyzer under a N_2_ atmosphere with a heating rate of 20 K/min. Moreover, a CHI760E electrochemical workstation was employed to realize the electrochemical impedance measurements of PSS/FP sensors, and impedance with frequencies ranging from 0.1 Hz to 1 MHz was determined under an amplitude of 0.01 V. A layer-by-layer self-assembled device was prepared for electrochemical testing. The device was obtained by encapsulating graphite paper and the innermost film sample with insulating foam. The electrochemical properties of the self-prepared multilayer electrochemical sensors were tested.

### 4.3. Preparation of PSS/FP Membranes

The PSS/FP membranes were prepared using a straightforward immersion method. Initially, the as-received PSS aqueous solution was diluted to 1%, 3%, 5%, 7%, and 9%. Then, FP membranes were immersed in various PSS solutions for 60 min and sonicated for 20 min to ensure complete impregnation. The impregnated PSS/FP membranes were then dried at 45 °C for 2 h for later use.

### 4.4. pH Responsiveness Test

Acid–base solutions with pH values of 1, 4, 7, 9, and 13 were prepared using 1 mol/L HCl solution and 1 mol/L NaOH solution. Then, 0.1 mL of these acid–base solutions with different pH values was dropped onto the device surface, and the stable current was measured using chronoamperometry.

### 4.5. Preparation of Artificial Sweat

First 1.0 g of urea, 5.0 g of sodium chloride, and 940 µL of lactic acid were placed into a 1000 mL beaker. Then, 900 mL of ultrapure water was added, and the pH was adjusted to 6.0 ± 0.2 using 0.1 mol/L HCl and NaOH. Then, the solution was transferred to a 1 L volumetric flask and diluted to a final volume of 1 L.

## Figures and Tables

**Table 1:** Loading dosages of PSS in PSS/FP membranes.

PSS Concentration	Loading Dosage
1%	0.2%
3%	6.7%
5%	15.4%
7%	12.4%
9%	20.9%

**Table 2:** PSS aqueous solution viscosity.

PSS Concentration	Viscosity (mPa.⋅S)
1%	248.9
3%	302.2
5%	337.8
7%	444.4
9%	613.3

## Data Availability

The data presented in this study are available upon request from the corresponding author.
